# Radical cystectomy in octogenarians: a propensity score-matched analysis of short-term outcomes

**DOI:** 10.1007/s00345-026-06513-y

**Published:** 2026-06-03

**Authors:** Maurin Helen Mangold, Luisa Egen, Nicolas Carl, Luisa Vivienne Renner, Alexander Studier-Fischer, Caelán Max Haney-Aubert, Maren Juliane Wenk, Maurice Stephan Michel, Niklas Westhoff, Karl-Friedrich Kowalewski

**Affiliations:** 1 Department of Urology and Urosurgery, University Medical Centre Mannheim, Medical Faculty Mannheim at Heidelberg University, Heidelberg, Germany; 2 German Cancer Research Centre (DKFZ) Heidelberg, Division of Intelligent Systems and Robotics in Urology (ISRU), Heidelberg, Germany; 3 DKFZ Hector Cancer Institute at the University Medical Centre Mannheim, Mannheim, Germany

**Keywords:** Radical cystectomy, Octogenarians, Mortality, Propensity score matching

## Abstract

**Purpose:**

This study aims to compare the impact of biological versus chronological age on postoperative risk after radical cystectomy (RC) by evaluating whether age ≥ 80 years independently predicts short-term mortality, complications and readmission rates beyond physiological status and comorbidity burden.

**Methods:**

We retrospectively analysed 879 patients undergoing open RC at a high-volume centre between 2015 and 2024. Propensity score matching (PSM; 1:1 nearest neighbor) was performed to balance octogenarians (80–89 years) and patients < 80 years for key covariates including ASA score, CCI score, BMI, renal function, preoperative albumin levels and urinary diversion. Primary endpoints were 30-day and 90-day mortality. Secondary endpoints included major complications (Clavien-Dindo ≥IIIb), 30- and 90-day readmission rates.

**Results:**

Among 879 patients, 114 (13%) were octogenarians. Primary analyses demonstrated a higher 30-day (6.1% vs. 1.4%; *p* = 0.003) and 90-day mortality (10.5% vs. 3.9%; *p* = 0.004) in octogenarians compared to patients < 80 years. After PSM (*n* = 194), mortality remained numerically higher in octogenarians (30-day: 6.2% vs. 2.1%; 90-day: 10.3% vs. 4.1%); however, these differences did not reach statistical significance (*p* = 0.28 and *p* = 0.165). Major complication and readmission rates were comparable between matched cohorts. In the multivariable regression analysis, age ≥ 80 years was not an independent predictor of any adverse outcome. Limitations include the retrospective design, single-centre setting and limited number of events after PSM.

**Conclusion:**

When comorbidity burden and physiological fitness are comparable, octogenarians undergoing RC achieve short-term outcomes similar to those of younger patients. Chronological age alone should not preclude consideration of RC. Individualised risk assessment, treatment in experienced, high-volume centres and most likely, increasing adoption of robot-assisted RC are essential for safe surgical care for older RC candidates.

**Supplementary Information:**

The online version contains supplementary material available at 10.1007/s00345-026-06513-y.

## Introduction

The demographic shift toward an ageing population in developed countries has profound implications for clinical practice. The number of adults aged 80 years and older worldwide is expected to triple by 2050 compared to 2015 [1]. Consequently, the management of older patients with complex medical conditions is becoming an increasingly significant challenge. Bladder cancer disproportionately affects the older population, with a median age at diagnosis of 73 years [2]. For patients with localised muscle-invasive bladder cancer (MIBC), radical cystectomy (RC) with or without perioperative chemotherapy and immunotherapy remains the standard of care (SOC) [3]. However, patients aged 80 years or older are substantially less likely to undergo RC than younger individuals [4]. This discrepancy reflects concerns regarding the substantial peri- and postoperative risks associated with RC in elderly patients. RC itself carries considerable morbidity and mortality risks, which increase with advancing age [5]. In octogenarians (80–89 years), 90-day mortality rates of up to 27% have been reported [5–8].

While chronological age remains an important predictor of postoperative mortality after RC, emerging evidence shows that functional status, comorbidities and physiological fitness may play a greater role in determining postoperative risk [5, 7]. To strengthen this evidence, we conducted a propensity-score-matched (PSM) analysis comparing postoperative outcomes between octogenarians and patients < 80 years undergoing RC at a high-volume centre. Our primary objective was to evaluate whether increased chronological age independently influences short-term mortality after adjustment for comorbidities and functional status as a surrogate for biological age. Secondary endpoints included major postoperative complications and readmission rates.

We aimed to evaluate whether well-selected octogenarians undergoing RC at a contemporary high-volume centre with standardised perioperative pathways demonstrate postoperative outcomes that are comparable to those of younger patients when adjusted for comorbidities and functional status, thereby challenging the conventional emphasis on chronological age. Such findings would strengthen the evidence base guiding preoperative RC counselling and shared decision-making in older patients.

## Materials and methods

### Patient population and outcomes

We retrospectively identified 879 patients aged ≥ 18 years who underwent RC for the treatment of muscle-invasive bladder cancer (MIBC) or high-risk non-muscle-invasive bladder cancer (NMIBC) between January 2015 and December 2024, using a prospectively maintained institutional database. Patients undergoing RC for non-oncological indications were excluded. Primary endpoints were 30-day and 90-day mortality. Secondary endpoints included complications during the initial hospital stay as well as 30-day and 90-day readmission rates. Clinicopathologic and perioperative variables were extracted from electronic health records (EHR), including American Society of Anesthesiologists (ASA) physical status and Charlson Comorbidity Index (CCI) [9, 10]. For descriptive and analytical purposes, CCI was dichotomised at > 5, a threshold supported by the comorbidity distribution in our cohort (median CCI among octogenarians: 6, IQR 5–7) and by prior data demonstrating significantly worse survival in patients with CCI > 5 after RC [11]. Complications were graded according to the Clavien-Dindo classification; major complications were defined as Clavien-Dindo (CDC) grade IIIb or higher [12, 13]. Readmissions were identified through institutional EHR. Vital status and mortality were verified via municipal registry inquiries in May 2025. All patients were managed according to our institutional Enhanced Recovery After Surgery (ERAS) protocol throughout the perioperative period. The study was approved by the Ethics Committee II of the Medical Faculty Mannheim of Heidelberg University (reference number 2024 − 800) and conducted in accordance with the Declaration of Helsinki.

### Statistical analysis

Octogenarians and patients < 80 years were balanced using PSM with a 1:1 nearest-neighbor method and a caliper of 0.2 standard deviations of the logit-transformed propensity score [14]. PSM covariates were specified a priori based on estimated clinical relevance and included sex, ASA, CCI, preoperative glomerular filtration rate (GFR), body mass index (BMI), preoperative albumin levels and continent vs. incontinent urinary diversion (UD) [15]. Covariate balance before and after PSM was assessed using standardised mean differences (see Supplementary Fig. 1). Continuous variables are reported as median [IQR] and categorical variables as frequency (%). Group comparisons were performed using t-tests or Mann-Whitney U tests for continuous variables and chi-square or Fisher’s exact tests for categorical variables. In both the unmatched and matched cohorts, univariable logistic regression models were used to examine the association between age group (≥ 80 vs. <80 years) and each outcome. Due to insufficient events in the matched cohort (*n* = 8), 30-day mortality was analysed using Fisher’s exact test instead of logistic regression. Multivariable logistic regression models were fitted in both the unmatched and matched cohorts to assess independent predictors of adverse outcomes. Following matching on UD type, complete separation occurred in the matched cohort. To ensure consistency across unmatched and matched models, UD type was not included as a covariate in the multivariable analyses. A p-value ≤ 0.05 was considered statistically significant. All analyses were conducted using R version 4.3.1 (RStudio; R Foundation for Statistical Computing, Vienna, Austria).

## Results

### Patient baseline characteristics

A total of 879 patients who underwent RC between January 2015 and December 2024 were included. The median age of the cohort was 69.00 [61.50, 76.00] years. Of these, 114 patients were octogenarians. Overall, 76.8% of the cohort were male. Compared to patients < 80 years, octogenarians had lower BMI (24.83 vs. 26.70; *p* = 0.001), lower preoperative GFR (48.00 vs. 67.00; *p* < 0.001), lower preoperative albumin (36.10 vs. 38.10; *p* < 0.001), higher ASA ≥ 3 rates (57.9% vs. 37.6%; *p* < 0.001) and higher comorbidity burden (CCI 6.00 vs. 3.00; *p* < 0.001). Muscle-invasive disease (≥ pT2) was more prevalent among octogenarians (77.2% vs. 58.3%; *p* < 0.001). Continent UD was performed significantly less frequently in the elderly cohort (2.6% vs. 49.0%; *p* < 0.001). After PSM, both cohorts were well balanced regarding baseline characteristics including GFR, BMI, ASA, CCI, preoperative albumin and UD. Details are described in Table 1.

**Table 1:** Clinicopathologic characteristics and postoperative outcomes of octogenarians (≥ 80 years) versus younger patients (< 80 years) in the overall, unmatched and propensity score-matched cohorts. Data are reported as median [IQR] or n (%).

	Overall	Unmatched		*p*-value	Matched		*p*-value
	*n* = 879	≥ 80 years *n* = 114	< 80 years *n* = 765		≥ 80 years *n* = 97	< 80 years *n* = 97	
Clinicopathologic characteristics							
Male Sex (%)	675 (76.8)	91 (79.8)	584 (76.3)	0.482	79 (81.4)	77 (79.4)	0.856
Age in years [IQR]	69.00 [61.50, 76.00]	83.00 [81.00, 85.00]	68.00 [60.00, 73.00]	**< 0.001**	83.00 [81.00, 85.00]	74.00 [70.00, 77.00]	**< 0.001**
BMI [IQR]	26.51 [23.66, 29.41]	24.83 [22.90, 27.63]	26.70 [23.88, 29.70]	**0.001**	24.86 [22.86, 27.68]	26.12 [22.86, 28.65]	0.408
Preoperative GFR [IQR]	64.00 [51.00, 80.00]	48.00 [39.00, 58.00]	67.00 [54.00, 83.00]	**< 0.001**	49.00 [39.00, 59.00]	53.00 [41.00, 63.00]	0.154
Preoperative Albumin [IQR]	38.00 [35.77, 40.00]	36.10 [34.00, 39.00]	38.10 [36.00, 40.10]	**< 0.001**	36.20 [34.00, 39.00]	37.50 [35.40, 39.00]	0.253
ASA ≥ 3 (%)	354 (40.3)	66 (57.9)	288 (37.6)	**< 0.001**	59 (60.8)	49 (50.5)	0.193
Total CCI [IQR]	4.00 [3.00, 5.00]	6.00 [5.00, 7.00]	3.00 [2.00, 5.00]	**< 0.001**	6.00 [5.00, 7.00]	5.00 [4.00, 7.00]	0.209
Diabetes (%)	186 (21.2)	29 (25.4)	157 (20.5)	0.282	23 (23.7)	36 (37.1)	0.061
CHD (%)	98 (11.1)	19 (16.7)	79 (10.3)	0.065	15 (15.5)	23 (23.7)	0.205
Urinary diversion (%)							
Continent	378 (43.0)	3 (2.6)	375 (49.0)	**< 0.001**	3 (3.1)	3 (3.1)	1.000
Incontinent	501 (57.0)	111 (97.4)	390 (51.0)		94 (96.9)	94 (96.9)	
T-Stage RC (%)							
< pT2	345 (39.2)	26 (22.8)	319 (41.7)	**< 0.001**	23 (23.7)	29 (29.9)	0.120
≥pT2	534 (60.8)	88 (77.2)	446 (58.3)		74 (76.3)	68 (70.1)	
N-Stage RC (%)							
N0	664 (75.5)	85 (74.6)	579 (75.7)	0.810	74 (76.3)	67 (69.1)	0.698
N+	201 (22.9)	26 (22.8)	175 (22.9)		22 (22.7)	28 (28.9)	
Nx	14 (1.6)	3 (2.6)	11 (1.4)		1 (1)	2 (2)	
R-Stage RC (%)							
R0	809 (92.0)	105 (92.1)	704 (92.0)	0.875	89 (91.8)	87 (89.7)	0.232
R1	56 (6.4)	8 (7.0)	48 (6.3)		8 (8.2)	9 (9.3)	
RX	14 (1.6)	1 (0.9)	13 (1.7)		0 (0)	1 (1.0)	
Postoperative outcomes							
CDC ≥ IIIb (%)	104 (11.8)	18 (15.8)	86 (11.2)	0.106	16 (16.5)	15 (15.5)	0.897
30-day readmission (%)	87 (9.9)	7 (6.1)	80 (10.5)	0.203	7 (7.2)	10 (10.3)	0.612
90-day readmission (%)	213 (24.2)	18 (15.8)	195 (25.5)	**0.033**	15 (15.5)	22 (22.7)	0.273
30-day mortality (%)	18 (2.0)	7 (6.1)	11 (1.4)	**0.003**	6 (6.2)	2 (2.1)	0.279
90-day mortality (%)	42 (4.8)	12 (10.5)	30 (3.9)	**0.004**	10 (10.3)	4 (4.1)	0.165

*RC* radical cystectomy, *GFR* glomerular filtration rate, *ASA* American Society of Anesthesiologists physical status classification, *CCI* Charlson Comorbidity Index, *CDC* Clavien–Dindo Classification, *CHD* coronary heart disease, *Continent diversion* neobladder/pouch, *Incontinent diversion* ileal conduit/ureterocutaneostomy

Bold p-values indicate statistical significance (p ≤ 0.05)

### Mortality rates

**Fig. 1:**
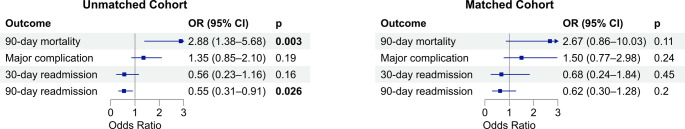
Forest plots displaying univariable logistic regression analysis of age ≥ 80 vs. age < 80 years on perioperative outcomes in the unmatched and propensity score-matched cohorts. Odds ratios (OR) with 95% confidence intervals (CI) are shown. Bold p-values indicate statistical significance (p ≤ 0.05). 30-day mortality is not displayed due to insufficient events in the matched cohort (n = 8) and is reported using Fisher’s exact test.

First, we evaluated mortality as a primary indicator of perioperative safety in patients ≥ 80 years undergoing RC. In our cohort, overall 30-day and 90-day mortality rates were 2% and 4.8%, respectively. Among unmatched patients, octogenarians had a significantly higher 30-day mortality (6.1% vs. 1.4%; *p* = 0.003) and 90-day mortality (10.5% vs. 3.9%; *p* = 0.004) compared to those < 80 years. Following PSM, no statistically significant difference was observed in either 30-day mortality (6.2% vs. 2.1%; *p* = 0.28) or 90-day mortality (10.3% vs. 4.1%; *p* = 0.17) between groups, although mortality rates remained numerically higher among octogenarians. In univariable analysis, advanced age ≥ 80 years was associated with an increased risk of 30-day (OR 4.48, 95% CI 1.62–11.64; *p* = 0.002) and 90-day mortality (OR 2.88, 95% CI 1.38–5.68; *p* = 0.003; Fig. 1) before matching. After PSM, this association was no longer statistically significant for either 30-day (Fisher’s exact: OR 3.11, 95% CI 0.54–32.33; *p* = 0.279) or 90-day mortality (OR 2.67, 95% CI 0.86–10.03; *p* = 0.11; Fig. 1). In summary, after PSM, mortality did not differ significantly between groups, despite numerically higher rates among octogenarians.

### Major complications and readmission rates

Next, major postoperative complications and readmission rates were compared between patients aged 80 years or older and those younger than 80 years to assess the potential impact of age on short-term postoperative outcomes. Overall, major complications occurred in 11.8% of patients. In the unmatched analysis, complication rates were 15.8% in patients aged ≥ 80 years versus 11.2% in those < 80 years (*p* = 0.106). After PSM, major complication rates were 16.5% and 15.5%, respectively (*p* = 0.897). Thirty-day readmission occurred in 9.9% overall, with rates of 6.1% in patients ≥ 80 years and 10.5% in those < 80 years (*p* = 0.203). After matching, 30-day readmissions were observed in 7.2% of octogenarians and 10.3% of patients < 80 years (*p* = 0.612). Ninety-day readmission was observed in 24.2% of patients overall. In the unmatched cohort, rates were 15.8% in patients aged ≥ 80 years and 25.5% in those aged < 80 years (*p* = 0.033). After matching, 90-day readmission rates were 15.5% and 22.7%, respectively (*p* = 0.273). Univariable analysis in the unmatched cohort did not show significant associations between age and major complications or 30-day readmission (major complications: OR 1.35, 95% CI 0.85–2.10; *p* = 0.19; 30-day readmission: OR 0.56, 95% CI 0.23–1.16; *p* = 0.16; Fig. 1). However, age ≥ 80 years was inversely associated with 90-day readmission (OR 0.55, 95% CI 0.31–0.91; *p* = 0.026; Fig. 1). No significant associations were found between age group and major complications or readmission rates in the matched cohort (major complications: OR 1.50, 95% CI 0.77–2.98; *p* = 0.24; 30-day readmission: OR 0.68, 95% CI 0.24–1.84; *p* = 0.45; 90-day readmission: OR 0.62, 95% CI 0.30–1.28; *p* = 0.2; Fig. 1). In summary, no differences in major complications or readmission rates were observed after adjustment for baseline characteristics, regardless of age (Fig. 1).

### Predictors of adverse outcomes

Group comparisons and univariable regression analyses were used to examine the relationship between age and postoperative outcomes. To assess the independent impact of age alongside other relevant risk factors, multivariable regression was performed in both the unmatched and matched cohorts. Due to an insufficient number of events, 30-day mortality could not be included as an outcome in the multivariable analysis.

**Fig. 2:**
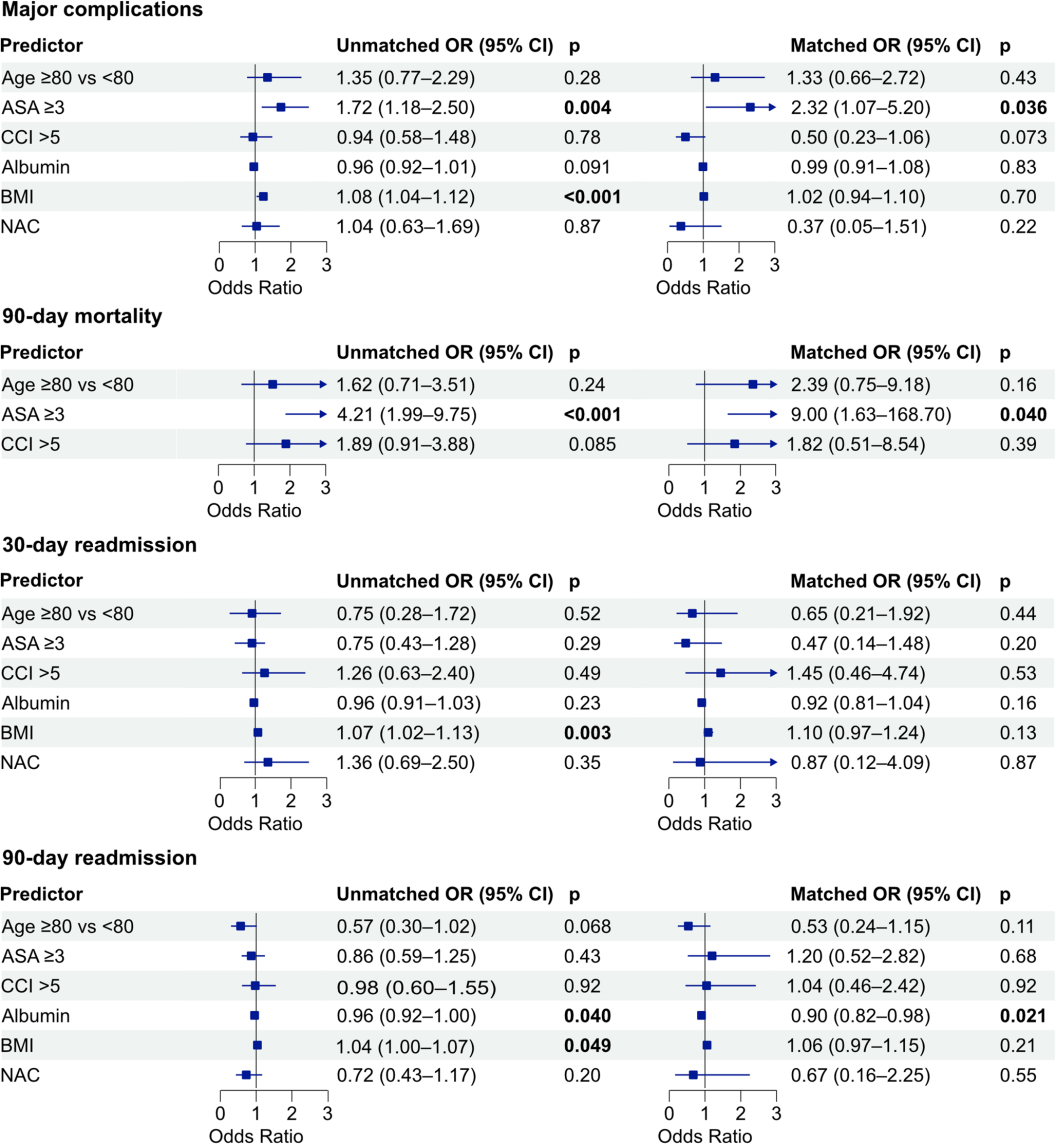
Forest plots displaying multivariable logistic regression analyses of predictors of postoperative outcomes in patients aged ≥ 80 vs. <80 years in the unmatched and propensity score-matched cohorts. Outcomes include major complications (Clavien-Dindo Classification [CDC] ≥ IIIb), 90-day mortality, 30-day readmission, and 90-day readmission. Covariates included age ≥ 80 years (reference: <80 years), American Society of Anesthesiologists (ASA) score ≥ 3, Charlson Comorbidity Index (CCI) > 5, preoperative serum albumin, body mass index (BMI), and neoadjuvant chemotherapy (NAC). Error bars indicate 95% confidence intervals. Bold p-values indicate statistical significance (p ≤ 0.05). 30-day mortality could not be included due to insufficient events.

 In the unmatched cohort, age ≥ 80 years was not independently associated with major complications (OR 1.35, 95% CI 0.77–2.29; *p* = 0.28), 90-day mortality (OR 1.62, 95% CI 0.71–3.51; *p* = 0.24), 30-day readmission (OR 0.75, 95% CI 0.28–1.72; *p* = 0.52), or 90-day readmission (OR 0.57, 95% CI 0.30–1.02; *p* = 0.068). In the matched cohort, age ≥ 80 years similarly showed no independent association with major complications (OR 1.33, 95% CI 0.66–2.72; *p* = 0.43), 90-day mortality (OR 2.39, 95% CI 0.75–9.18; *p* = 0.16), 30-day readmission (OR 0.65, 95% CI 0.21–1.92; *p* = 0.44), or 90-day readmission (OR 0.53, 95% CI 0.24–1.15; *p* = 0.11) (Fig. 2).

Regarding other predictors, higher ASA classification (≥ 3) was significantly associated with major complications in both the unmatched (OR 1.72, 95% CI 1.18–2.50; *p* = 0.004) and matched cohorts (OR 2.32, 95% CI 1.07–5.20; *p* = 0.036), as well as with 90-day mortality in both the unmatched (OR 4.21, 95% CI 1.99–9.75; *p* < 0.001) and matched cohorts (OR 9.00, 95% CI 1.63–168.70; *p* = 0.040), with the latter to be interpreted cautiously given the wide confidence interval. BMI was independently associated with major complications in the unmatched cohort (OR 1.08, 95% CI 1.04–1.12; *p* < 0.001), as well as with both 30- and 90-day readmission in the unmatched cohort (30-day: OR 1.07, 95% CI 1.02–1.13; *p* = 0.003; 90-day: OR 1.04, 95% CI 1.00–1.07; *p* = 0.049). Higher albumin levels were independently associated with lower odds of 90-day readmission in the matched cohort (OR 0.90, 95% CI 0.82–0.98; *p* = 0.021) (Fig. 2).

In summary, after adjustment for key clinical variables, age was not an independent predictor of any adverse outcome in our cohort. ASA was the most consistent independent predictor, associated with both major complications and 90-day mortality, while albumin and BMI were associated with readmission outcomes

## Discussion

 The aim of this study was to clarify whether chronological age independently affects postoperative outcomes after RC. Using PSM to balance comorbidity burden and physiological status, we found that octogenarians treated at a high-volume RC centre did not experience significantly higher short-term mortality, major complications or readmissions than matched younger patients < 80 years. Our findings support that, in well-selected cases, age ≥ 80 years alone should not preclude consideration for potentially curative RC.

 A key finding of our analysis is that comorbidity burden and functional status seem to provide greater prognostic value for postoperative mortality than chronological age alone. Although octogenarians exhibited substantially higher 30- and 90-day mortality in the unmatched analysis, these differences were attenuated after matching for ASA score, comorbidity burden, BMI, renal function, albumin levels and UD type. Importantly, mortality rates remained numerically higher among octogenarians after matching, and age-related risk may therefore not be eliminated by adjustment for baseline characteristics. In the multivariable analysis of the matched cohort, age ≥ 80 years was not identified as an independent predictor of short-term mortality; however, the wide confidence intervals highlight limited statistical precision due to the small number of events and preclude definitive conclusions regarding residual age-related risk.

 The predictive value of clinical risk factors, such as higher comorbidity burden and reduced functional status, on mortality is well supported by the literature: Schulz et al. reported nearly two-fold higher 90-day mortality (7.6% vs. 3.2%) and CDC grade ≥ 3 complications (14.6% vs. 9.4%) among patients with ASA ≥ 3 compared with those with ASA < 3 [16]. Regarding comorbidity, Koppie et al. demonstrated a clear survival gradient across age-adjusted CCI scores, with median overall survival decreasing from 6.3 years in RC patients with low comorbidity (CCI ≤ 2) to 1.7 years in those with high comorbidity (CCI > 5) [11]. Extending this concept, more recent work has focused on multidimensional frailty assessment: Duwe et al. showed that frailty indices and geriatric assessment tools including the simplified frailty index (sFI) and Adult Comorbidity Evaluation 27 score (ACE-27) outperform chronological age in discriminating postoperative risk including mortality; however, in contrast to our findings, age ≥ 80 years remained an independent predictor of 90-day mortality in their cohort [6]. Beyond single-centre geriatric assessments, a recent population-based analysis using a claims-based frailty index (CFI) in over 6000 patients undergoing RC aged 66 and older demonstrated that increasing frailty was independently associated with higher 90-day morbidity, readmissions and mortality [17]. Corroborating this across procedural contexts, the modified frailty index (mFI) has demonstrated independent predictive value beyond age alone even in lower-complexity urological interventions, reinforcing that frailty, rather than chronological age, should guide surgical candidacy in this population [18].

Complementing frailty-based approaches, preoperative laboratory biomarkers have emerged as additional tools for perioperative risk stratification. Preoperative anaemia, hyperfibrinogenaemia, and poor nutritional status, as assessed by the Controlling Nutritional Status (CONUT) score, a composite of serum albumin, total lymphocyte count, and cholesterol, have been independently associated with major complications and worse oncological outcomes after RC, and should be addressed in prehabilitation, particularly in high-risk elderly patients [19, 20]. Consistent with this, preoperative serum albumin emerged as an independent predictor of 90-day readmission in our cohort, further supporting its role as a clinically relevant marker of perioperative risk in this population.

 Overall, the 90-day mortality rates observed in our cohort compare favourably with contemporary studies in older populations, most of which report 90-day mortality between 11% and 14% [5, 8, 21]. Regarding these comparatively low rates, treatment at experienced high-volume centres appears particularly important for older patients. A large analysis of more than 12,000 RC patients, including over 1,600 octogenarians, demonstrated that the majority of elderly patients (81.7%) between 1998 and 2007 were treated in low- or intermediate-volume hospitals where inpatient mortality and perioperative complications remained significantly higher in octogenarians even after PSM [22]. Although centralisation of RC to specialised centres has likely increased since that period, these real-world data highlight the importance of institutional volume and expertise in providing RC to elderly patients. Consistent with this, Zakaria et al. reported substantially lower mortality in elderly patients when RC was performed in academic centres compared to community hospitals (9% vs. 16.3%; *p* < 0.001) [8]. We believe that our low mortality rates also underscore the importance of specialised high-volume centres in providing safe surgical care for elderly RC candidates, including those with substantial comorbidity burdens. In addition, growing evidence supports that robot-assisted RC (RARC) is associated with lower perioperative mortality and fewer major complications in elderly patients compared to open RC [23, 24]. A prospective multicentre cohort demonstrated significantly lower blood loss, blood transfusions and shorter hospital stay with RARC with ureterocutaneostomy (UCS) compared to open or laparoscopic RC with UCS in octogenarians [25]. Similarly, a single-centre retrospective analysis found comparable overall complication rates between elderly and younger RARC patients; however, postoperative ileus occurred significantly more frequently in octogenarians and prolonged hospitalisation, underscoring the importance of ERAS-guided perioperative management [26]. Our findings must therefore be contextualised within the evolving surgical landscape. The results reported in our cohort based exclusively on open RC may represent a conservative estimate, with potential for further improvement as minimally invasive approaches become more widely adopted in this population.

 For patients in whom RC carries excessive perioperative risk despite optimal centre selection and robotic approaches, bladder-preserving surgery may represent a viable oncological alternative. Partial cystectomy with or without pelvic lymph node dissection has been shown to yield comparable overall survival in octogenarians with localised MIBC [27].

 Our findings also highlight the substantial morbidity associated with RC, regardless of patient age. Across all stratifications, rates of major complications in our study ranged from 11.2% to 16.5%. This underlines the inherent procedural risk of RC and aligns with prior large-scale reports [28–30]. Nevertheless, complication rates did not differ between age groups after matching, and ASA ≥ 3, but not age ≥80 years, was the independent predictor of major complications. Thus, major morbidity after RC appears to reflect physiological burden more than the patient’s chronological age.

Beyond perioperative morbidity, postoperative recovery and functional outcomes are particularly relevant in elderly RC candidates and should be addressed during preoperative counselling. Even in the absence of higher short-term complication rates, older patients may experience prolonged recovery and delayed return to baseline functional status. In a large multicentre cohort of adults aged ≥ 80 years undergoing inpatient non-orthopaedic surgery, Zhang et al. reported that approximately one in five patients experienced persistent functional decline at 30 days, reflected by new or increased dependency in activities of daily living [31]. These data underscore that shared decision-making in octogenarians should incorporate not only perioperative risk estimates but also the possibility of prolonged functional impairment and rehabilitation needs.

 Finally, when discussing RC in octogenarians, demographic trends must be considered. Recent epidemiological studies have shown that frailty indices among adults aged 75–90 continue to decline, with cohorts becoming generally fitter over time [32]. Improvements in functional health across ageing cohorts suggest steady gains in physical reserve and overall physiological robustness [33]. These observations highlight the need for periodic reassessment of RC risk in contemporary octogenarians, whose comorbidity profiles will be evolving substantially over time.

 This study is not free of limitations. First, the retrospective, single-centre design limits generalisability and carries inherent risk of selection bias. Additionally, as a high-volume centre performing more than 100 RCs annually, our results may not be representative of lower-volume institutions. Furthermore, although our initial cohort was large, the number of events after matching was low, substantially reducing statistical power, particularly for mortality comparisons. The resulting wide confidence intervals indicate considerable uncertainty around point estimates, and clinically relevant differences cannot be ruled out despite non-significant results. Moreover, the lack of frailty assessment and detailed functional status data precluded evaluation of modern multidimensional risk tools, which may have offered additional predictive value. However, key components of established frailty indices are substantially captured by ASA and CCI, which we consider reasonable but imperfect surrogates for validated geriatric instruments such as the modified Frailty Index (mFI) or Comprehensive Geriatric Assessment (CGA). Future prospective studies should incorporate these tools to better stratify surgical risk in octogenarian candidates. Furthermore, given that both matched groups consisted almost exclusively of patients receiving incontinent diversion, findings from the matched analysis cannot be extrapolated to patients undergoing continent diversion. In the unmatched cohort, the marked imbalance in diversion type represents a potential confounder that cannot be fully excluded when interpreting outcome differences between age groups. Additionally, findings apply primarily to octogenarians matched against younger patients with comparably elevated comorbidity burden and may not be generalisable to the broader RC population. Finally, our cohort comprised exclusively patients treated with open RC, which limits direct applicability to contemporary practice where RARC is increasingly adopted and has demonstrated superior perioperative outcomes in elderly cohorts. Given the increasing adoption of RARC, future studies that integrate both open and robotic approaches in contemporary octogenarian populations will be critical for refining risk estimates and supporting clinical decision-making in older RC patients.

## Conclusion

 In conclusion, octogenarians treated at our high-volume centre showed a comparatively low perioperative mortality rate (30-day: 6.1%; 90-day: 10.5%). After matching for comorbidity burden and preoperative fitness, differences in mortality were attenuated and no longer statistically significant. However, numerically higher mortality rates persisted among octogenarians, and clinically relevant differences cannot be excluded given the limited number of events and wide confidence intervals in the matched analysis. These findings indicate that chronological age alone provides limited prognostic information when considered in isolation, whereas physiological reserve and comorbidity burden appear to be the primary determinants of postoperative risk. These findings support offering RC to carefully selected elderly patients and highlight the importance of high-volume centres in providing surgical care for this growing patient population.

## Supplementary Information

Below is the link to the electronic supplementary material.

Supplementary Material 1

## Data Availability

The data that support the findings of this study are available from the corresponding author upon reasonable request. Due to the sensitive nature of the patient data, access is restricted to ensure confidentiality and compliance with ethical standards.
