# Supplementary material for: Radical cystectomy in octogenarians: a propensity score-matched analysis of short-term outcomes
Source: World J Urol. 2026 Jun 3;44(1):405. doi: 10.1007/s00345-026-06513-y (PMC13233869; doi:10.1007/s00345-026-06513-y)
Supplement: Supplementary file 1 — Supplementary Material 1 [file 345_2026_6513_MOESM1_ESM.docx]

**Supplementary Figure 1:** Standardised mean differences of baseline covariates between octogenarians (≥80 years) and younger patients (<80 years) before (unadjusted) and after propensity score matching (PSM, adjusted). PSM substantially reduced imbalances across all included covariates (sex, BMI, GFR, ASA score, total CCI score, preoperative albumin and urinary diversion).
